# Analysis of vertebrate genomes suggests a new model for clade B serpin evolution

**DOI:** 10.1186/1471-2164-6-167

**Published:** 2005-11-23

**Authors:** Dion Kaiserman, Phillip I Bird

**Affiliations:** 1 Department of Biochemistry & Molecular Biology, Monash University, Clayton, Victoria, Australia

## Abstract

**Background:**

The human genome contains 13 clade B serpin genes at two loci, 6p25 and 18q21. The three genes at 6p25 all conform to a 7-exon gene structure with conserved intron positioning and phasing, however, at 18q21 there are two 7-exon genes and eight genes with an additional exon yielding an 8-exon structure. Currently, it is not known how these two loci evolved, nor which gene structure arose first – did the 8-exon genes gain an exon, or did the 7-exon genes lose one? Here we use the genomes of diverse vertebrate species to plot the emergence of clade B serpin genes and to identify the point at which the two genomic structures arose.

**Results:**

Analysis of the chicken genome indicated the presence of a single clade B serpin gene locus, containing orthologues of both human loci and both genomic structures. The frog genome and the genomes of three fish species presented progressively simpler loci, although only the 7-exon structure could be identified. The *Serpinb12 *gene contains seven exons in the frog genome, but eight exons in chickens and humans, indicating that the additional exon evolved in this gene.

**Conclusion:**

We propose a new model for clade B serpin evolution from a single 7-exon gene (either *Serpinb1 *or *Serpinb6*). An additional exon was gained in the *Serpinb12 *gene between the tetrapoda and amniota radiations to produce the 8-exon structure. Both structures were then duplicated at a single locus until a chromosomal breakage occurred at some point along the mammalian lineage resulting in the two modern loci.

## Background

The serpins are a superfamily of proteins sharing a conserved tertiary structure [1] that has evolved primarily to control proteolytic activity, although some have evolved non-inhibitory functions [2-6]. To date, over 500 serpin sequences have been identified in the genomes of species from almost all phyla including viruses, bacteria, metazoans and plants [1,7].

The serpin mechanism of inhibition is based on the metastable nature of the native serpin fold [8]. The reactive centre loop (RCL) is exposed at the top of the serpin where it acts as a pseudosubstrate for the target proteinase [9]. Following cleavage of the RCL, a conformational rearrangement, termed the stressed to relaxed transition, occurs and the RCL inserts into a β-sheet forming an extra β-strand [10]. This rearrangement is responsible for the distortion and irreversible inactivation of the target proteinase [11].

Phylogenetic analysis has divided serpins into 16 clades and a number of orphan sequences [1]. The clade B serpins are primarily intracellular proteinase inhibitors [12]. In humans this clade consists of 13 members and includes regulators of inflammation, apoptosis and angiogenesis (reviewed in [13]). The genes are arranged at two genomic loci at 6p25 (3 genes: *SERPINB1*, *SERPINB6 *and *SEPINB9*) and 18q21 (10 genes: *SERPINB2*, *B3*, *B4*, *B5*, *B7*, *B8*, *B10*, *B11*, *B12 *and *B13*), and all clade B serpins described to date conform to two related gene structures of seven or eight exons. Both the phasing and positioning of the intron/exon splice sites are identical for six introns, leading to conserved exon lengths. The additional intron splice site of the 8-exon structure is also conserved, although the extra exon encodes a highly divergent sequence of variable length, termed the CD loop.

Although clade B serpins have been identified in mammals (humans, mice and rats) as well as birds, it is not known how they evolved, nor has the primordial structure or gene been identified. Two competing models exist to explain the presence of two clade B loci in humans. The first proposes duplication of the entire 6p25 locus followed by a number of single gene duplications at 18q21 [14]. The second proposes duplication of a single gene from 18q21 to 6p25, followed by successive duplications at both loci to derive the modern complement [15]. Two key differences are apparent between these models. The former assumes that the 7-exon structure and 6p25 locus are ancestral, whereas the latter assumes the opposite.

Here, we use the completed chicken genome to show that the two mammalian loci are the result of an ancient chromosomal split, as orthologues of genes from both human loci are linked in the chicken. Further analysis of emerging genome sequences suggests that clade B serpin genes arose from a primordial *Serpinb1 *or *b6 *gene with seven exons, the additional exon being gained in *Serpinb12 *after the divergence of amphibians and amniotes.

## Results

### Clade B serpin genes in the chicken (*Gallus gallus*)

To determine which of the two clade B serpin loci is ancestral, the chicken genome was investigated, as it represents a distantly related species. This analysis was also recently performed independently by another group, yielding similar results [16]. As illustrated in Figure 2A, the chicken genome contains a single clade B serpin locus on chromosome 2, composed of 10 genes, nine of which are supported by EST data. Both gene structures are also evident within the locus. Single orthologues exist in the chicken for the 7-exon human genes *SERPINB1 *(MNEI), *SERPINB5 *(maspin) and *SERPINB6 *(PI6), and also for the 8-exon genes *SERPINB2 *(PAI-2) and *SERPINB12 *(yukopin). Human *SERPINB10 *(bomapin), an 8-exon gene, has 2 chicken homologues – *MENT *(which fits the 8-exon arrangement) and a MENT-like gene for which the structure cannot be completely determined. The three remaining chicken genes *Serpinb14 *(ovalbumin), *Serpinb14b *(geneY) and *Serpinb14c *(geneX) are highly related [17] and have no human orthologues. This suggests that the common ancestor of chickens and mammals had a complement of six clade B serpin genes: *SERPINB1*, *B2*, *B5*, *B6*, *B10 *and *B12*. Furthermore, the organization of the chicken clade B serpin locus matches that of humans, contradicting both previously proposed models of clade B serpin gene evolution. Rather than two independently evolving loci, it suggests that both the 7- and 8-exon gene structures evolved at a single locus in the common ancestor of birds and mammals that then split to yield the 2 loci apparent in humans and rodents.

**Figure 1:**
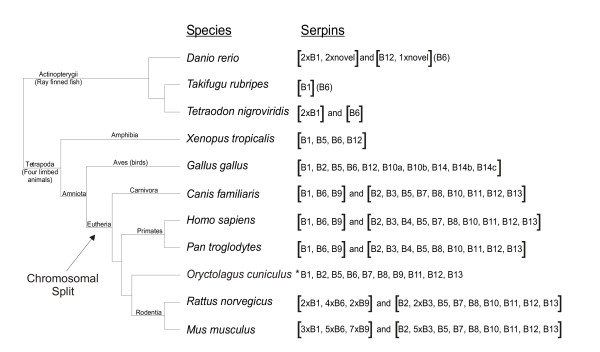
**Distribution of clade B serpin genes in vertebrate genomes**. A dendrogram showing relationships between vertebrate species with clade B serpin gene loci. The genes identified within each genome are indicated on the right without the *SERPIN *root designation. Multiple loci are delineated by square brackets, while genes identified in EST databases, but not in the genome, are in round brackets. * due to the incomplete nature of the sequencing data, the structure of the rabbit (*Oryctolagus cuniculus*) loci could not be established. Branch lengths are not to scale.

**Figure 2:**
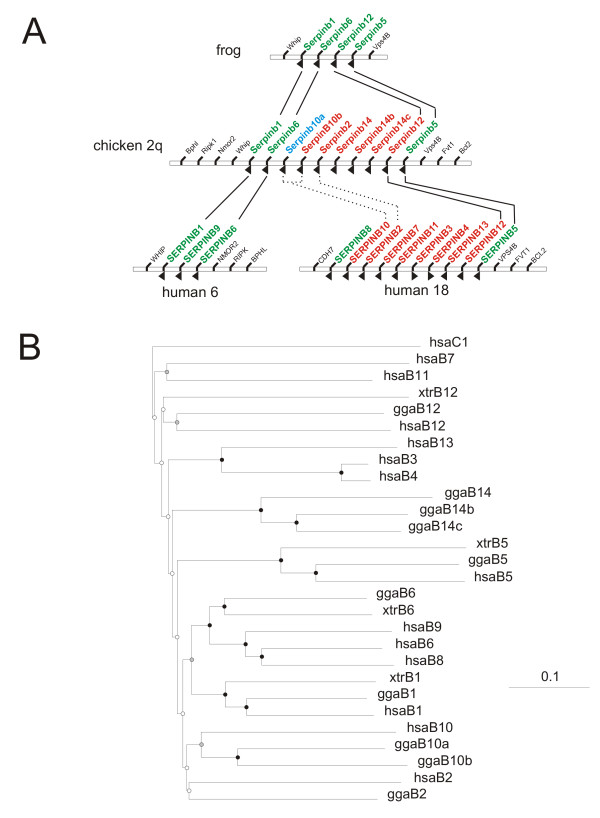
**Comparison of frog, chicken and human clade B serpin loci**. The structure of the serpin locus in frog, chicken and human is shown with 7-exon serpin genes in green, 8-exon serpin genes in red and non-serpin genes in black. Gene structure could not be predicted for *Serpinb10a *(blue). Arrows indicate the direction of transcription. Orthologous genes with strong RCL conservation are linked with solid lines, dotted lines denote inter-species orthologues with weak RCL homology. (B) The amino acid sequences of human, chicken and frog clade B serpins were aligned with human antithrombin (*SERPINC1*) and a Neighbour-Joining tree constructed (gapped positions removed, 1000 bootstraps) to show evolutionary relationships. Scale bar indicates the number of substitutions at each site. Node colour indicates the bootstrap value (black >75%, grey 50–75%, white <50%). Genes are named without the *SERPIN *root. hsa = *Homo sapiens*; gga = *Gallus gallus*; xtr = *Xenopus tropicalis*.

### Evolution of clade B serpin genes in vertebrates

Since both the 7 and 8-exon gene structures were present in the chicken genome, we investigated more distantly related genomes to identify which structure arose first. Each of the 13 human protein sequences were used to probe the current builds of vertebrate genomes to identify clade B serpin genes. This identified genes with strong RCL similarity to human clade B serpins in the genomes of fish, amphibians and various mammals. By plotting the presence of genes on a dendrogram of species a pattern of gene evolution can be proposed (Figure 1).

As described above, the common ancestor of mammals and birds contained a single clade B serpin gene locus of six genes (*Serpinb1*, *b2 b5*, *b6*, *b10 *and *b12*). All of the mammalian genomes examined so far (human, chimpanzee, dog, rat and mouse) contain two loci, suggesting that the chromosomal split occurred early in mammalian evolution (Figure 1). The locus syntenic with human 6p25 contains *SERPINB1*, *B6 *and *B9 *in humans, chimpanzees, and dogs and an expanded repertoire of 15 and 8 in mice and rats, respectively [18,19], indicating further expansion within the rodent lineage. The incomplete rabbit genome yielded hits for 10 of the human clade B serpins (*Serpinb3*, *b4 *and *b10 *were not identified), with no evidence of the expansion observed in rats or mice. Furthermore, the *Serpinb9 *RCL sequence of rabbits is predicted to match that of humans whereas the rodent *Serpinb9 *RCL is mutated.

The locus syntenic to human 18q21 is identical between mammals with the exception of the *SERPINB3 *and *SERPINB4 *genes (SCCA-1 and -2), which are unique to each species (data not shown and [20]). This suggests that 12 genes were present in the mammalian common ancestor – *SERPINB1*, *B6 *and *B9 *at one locus, and *SERPINB2*, *B5*, *B7*, *B8*, *B10*, *B11*, *B12*, *B13 *and the primordial *SERPINB3/B4 *at the other. Since then, the ancestral *SERPINB3/B4 *has evolved independently in each mammalian species.

Further back in evolution, chickens and mammals shared a common ancestor with amphibians approximately 300 million years ago, and the genome of *Xenopus tropicalis *(the western clawed frog) provides an insight into this more primitive gene cluster (Figure 1). The *X. tropicalis *genome contains a single clade B serpin locus of 4 genes (*Serpinb1*, *Serpinb6*, *Serpinb5 *and *Serpinb12*), bounded by orthologues of *WHIP *and *VPS4B*. The placement of both serpin and non-serpin genes is conserved between the frog and chicken genomes, strengthening our hypothesis that a single clade B serpin gene cluster has split at some point in mammalian evolution (Figure 2A). Again, the phylogenetic tree confirms that clade B serpin genes cluster on the basis of conserved function, rather than by species (Figure 2B).

450 million years ago, amphibians, birds and mammals shared a common ancestor with fish [21]. Three fish genomes were available for analysis: *Danio rerio *(zebrafish)*, Tetraodon nigroviridis *(green pufferfish) and *Takifugu rubripes *(japanese pufferfish). The *T. rubripes *and *T. nigroviridis *genomes each contain one or two copies of *Serpinb1*, respectively, with no other serpin genes for over 100 kb upstream or downstream, while the *D. rerio *locus contains two copies of *Serpinb1*, linked to two other genes with RCL sequences that do not match any human serpins. However, ESTs derived from both *Serpinb1 *and *Serpinb6 *can be identified from all three species, indicating the presence of another clade B serpin locus. Indeed, a second locus is identifiable in the *D. rerio *and *T. nigroviridis *genomes. The *T. nigroviridis *locus contains only *Serpinb6*, although the contig is very short and other genes may exist. The *D. rerio *region contains a number of clade B serpin gene fragments as well as a gene with RCL homology to human *SERPINB12*, although no orthologue of *SERPINB6 *can be identified. However, the BAC clone finishes in intronic sequence between the final two exons of a serpin gene, and therefore this fragment may be the 5' region of *D. rerio Serpinb6*.

### Gene structure

The gene structures of the newly identified genes from Xenopus and fish were determined by alignment of ESTs to genomic sequence as well as by GENSCAN predictions (Table 2). Human *SERPINB1 *is a 7-exon gene, and this is also the case for *Serpinb1 *in the three fish genomes investigated, as well as the novel genes identified in *Danio rerio*. Lack of EST coverage resulted in an inability to determine intron phasing for the *T. nigroviridis *and *T. rubripes *genes, however, the positioning of the introns within the predicted mRNA was consistent with the mammalian clade B serpins.

**Table 1:** Accession numbers of sequences used in this study

**Gene (Species)**	**Accession Number(s)**
*Serpinb1 (Oncorhynchus mykiss)*	[Genbank:CX143380]
*Serpinb6 (Oncorhynchus mykiss)*	[Genbank:AY606039]
*Serpinb1 (Salmo salar)*	[Genbank:CA059107; Genbank:CA051086]
*Serpinb6 (Salmo salar)*	[Genbank:CK877893]
*Serpinb1 (Oryzias latipes)*	[Genbank:BJ721045]
*Serpinb6 (Oryzias latipes)*	[Genbank:BJ530676]
*Serpinb6 (Ictalurus punctatus)*	[Genbank:CK420790]
*Serpinb1 (Haplochromis)*	[Genbank:BJ692672]
*Serpinb1 (Gasterosteus aculeatus)*	[Genbank:CD504030]
*Serpinb1 (Danio rerio)*	[Genbank:AL912530]
*Serpinb6 (Danio rerio)*	[Genbank:CD015363]
*Serpinb1 (Xenopus tropicalis)*	[Genbank:CX372321; Genbank:CF593092; Genbank:CX383158; Genbank:DR857859]
*Serpinb5 (Xenopus tropicalis)*	[Genbank:CX847450; Genbank:CF346211]
*Serpinb6 (Xenopus tropicalis)*	[Genbank:BX709151; Genbank:DR860385; Genbank:DN093291]
*Serpinb12 (Xenopus tropicalis)*	[Genbank:DR880497]
*Serpinb1 (Gallus gallus)*	[Genbank:CD216625; Genbank:CF255528; Genbank:CF257148]
*Serpinb2 (Gallus gallus)*	[Genbank:BU409199; Genbank:BU205882; Genbank:BU410099]
*Serpinb5 (Gallus gallus)*	[Genbank:CD739917]
*Serpinb6 (Gallus gallus)*	[Genbank:CF250725; Genbank:BU116697; BU133732; Genbank:AJ734077]
*Serpinb12 (Gallus gallus)*	[Genbank:BU442268]

**Table 2:** Intron/exon phasing in vertebrate clade B serpin genes

**Species**	**Gene^1^**	**A^2^**	**B**	**C^3^**	**D**	**E**	**F**	**G**
*D. rerio*	B1	UTR	0	NP	0	1	0	0
*T. nigroviridis*^†^ *T. rubripes*^†^	B1	UTR	0	NP	0	1	0	0
*X. tropicalis*	B1	UTR	0	NP	0	1	0	0
	B5	UTR	0	NP	0	1	0	0
	B6	UTR	0	NP	0	1	0	0
	B12	UTR	0	NP	0	1	0	0
*G. gallus*	B1	UTR	0	NP	0	1	0	0
	B2	UTR	0	0	0	1	0	0
	B5	UTR	0	NP	0	1	0	0
	B6	UTR	0	NP	0	1	0	0
	B10a	UTR	0	*	0	1	0	0
	B10b	UTR	0	0	0	1	0	0
	B12	UTR	0	0	0	1	0	0
	B14	UTR	0	0	0	1	0	0
	B14b	UTR	0	0	0	1	0	0
	B14c	UTR	0	0	0	1	0	0
*H. Sapiens*	B1	UTR	0	NP	0	1	0	0
	B2	UTR	0	0	0	1	0	0
	B3/4	UTR	0	0	0	1	0	0
	B5	UTR	0	NP	0	1	0	0
	B6	UTR	0	NP	0	1	0	0
	B7	UTR	0	0	0	1	0	0
	B8	UTR	0	NP	0	1	0	0
	B9	UTR	0	NP	0	1	0	0
	B10	UTR	0	0	0	1	0	0
	B11	UTR	0	0	0	1	0	0
	B12	UTR	0	0	0	1	0	0
	B13	UTR	0	0	0	1	0	0

^1 ^Gene names are given without the Serpin root.

^2 ^UTR = intron occurs in the 5' untranslated region and thus has no phase

^3 ^NP = Intron not present, * = Intron cannot be reliably predicted and no EST exists.

^† ^Intron phasing predicted from consensus sequence

Overlapping ESTs allowed construction of complete mRNA sequences for all 4 of the Xenopus genes. *Serpinb1*, *b5 *and *b6 *conformed to the 7-exon structure, and both positioning and phasing of the intron/exon boundaries was conserved with humans. The other gene to appear in frogs is *Serpinb12*, an 8-exon gene in the chicken and mammalian genomes. However, this gene in frogs contained only 7-exons, lacking the extra exon encoding the CD loop. This gene structure was confirmed by EST DR880497 which spans all 6 intron/exon boundaries. As shown in Figure 2B, Xenopus *Serpinb12 *is clearly related to human and chicken *Serpinb12*, suggesting that the 8-exon structure arose some time between the divergence of amphibians and amniotes in the *Serpinb12 *gene.

## Discussion

The human genome contains two clusters of clade B serpin genes at 6p25 and 18q21. The 13 genes all conform to either a 7- or 8-exon gene structure with conservation of both phasing and positioning of intron/exon boundaries. To address possible mechanisms of clade B gene evolution, the chicken genome was analysed and found to contain a single clade B gene cluster of 10 genes. Another recent investigation of the chicken genome also identified these 10 genes, however, the authors concluded that the newly identified MENT-like chicken gene, *Serpinb10a*, and human *SERPINB10 *represented direct orthologues [16]. This was based on the overall protein similarity as well as homology within the CD loop and RCL. While the RCL sequence of chicken *Serpinb10a *and human *SERPINB10 *have some similarities, there are a number of charge differences throughout the RCL, and optimal alignment requires insertion of a gap at the P side (N-terminal to the point of proteolytic cleavage), a feature not observed between other chicken-human orthologues. Furthermore, although the presence of a CD loop would be predicted in *Serpinb10a *given its evolutionary ancestry, the presence of an eighth exon requires experimental proof. Without direct sequencing of a transcript, it remains possible that the exon may be missing or contain a premature stop codon or mis-sense mutation resulting in a lack of functional protein expression. The exon identified by Benarafa and Remold-O'Donnell had a very low statistical score [16], and decreasing the GENSCAN settings to detect this exon leads to the identification of a number of potential exons in other serpin genes that are not transcribed (data not shown).

**Figure 3:**
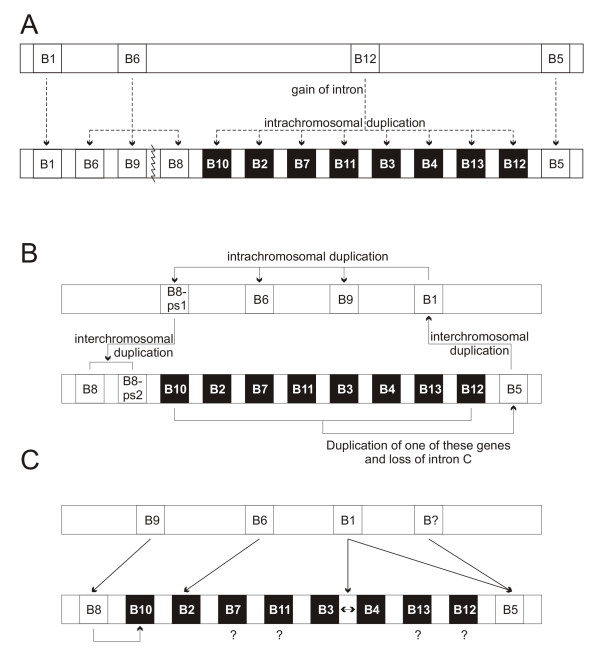
**A new mechanism of clade B serpin gene expansion**. (A) A new model for evolution of two clade B serpin loci in mammals, from a single locus of 4 genes. A series of intrachromosomal duplications results in 13 genes, followed by chromosomal breakage to yield two loci. Two previously proposed mechanisms based on duplication of an ancestral locus as described by (B) Bartuski *et al*. [14]; and (C) Scott *et al*. [15]. Arrows represent gene duplication events. Black boxes indicate genes displaying the 8-exon structure, white boxes are 7-exon genes. Genes are named without the *SERPIN *root. Diagram not to scale.

The identification of orthologues (matching RCLs) of both mammalian clusters (*SERPINB2*, *SERPINB5 *and *SERPINB12 *from human 18q21 and *SERPINB1 *and *SERPINB6 *from human 6p25) at a single genomic locus in the chicken raises the intriguing possibility that the serpin clusters arose from an ancient chromosomal breakage, rather than chromosomal duplication events. More distantly related genomes appear to support this conclusion as a single clade B serpin gene cluster exists in the *Xenopus tropicalis *genome, consisting of *Serpinb1*, *b5*, *b6 *and *b12*. Therefore, the two loci observed in mammals arose from this single cluster of four 7-exon genes. *Serpinb12 *gained an extra exon and was duplicated to give rise to the 8-exon genes, followed by a chromosomal split yielding two loci. This presents a much simpler mechanism for the observation of different genetic structures at a single locus than previously proposed models [14,15] (Figure 3).

**Figure 4:**
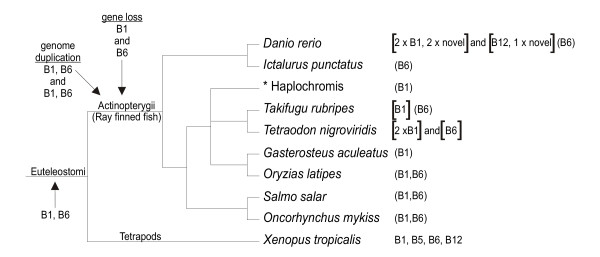
**Distribution of clade B serpin ESTs in fish**. A dendrogram of fish species is shown with clade B serpins ESTs shown on the right in brackets. The tetrapoda lineage is also indicated, with the simplest locus (*Xenopus tropicalis*) shown for comparison. Genes are named without the *SERPIN *root. The relative timing of genome duplication and subsequent gene loss within ray finned fish are shown as well as the composition of the loci after each event. Asterisk indicates an EST for which only the genus name was given.

Since *Serpinb1 *and *Serpinb6 *are linked in frogs, chickens and humans, it is likely that they were linked in their common ancestor with fish, however, this was not the case in any of the fish genomes investigated. This may be due to a whole genome duplication that occurred early in the evolution of ray finned fish, followed by rapid loss of duplicate genes [22]. This could give rise to the two serpin loci that have since evolved separately (Figure 4). Therefore, we propose that the single primordial serpin clade B locus (in the common ancestor of fish and tetrapods) contained only *Serpinb1 *and *Serpinb6*, as these are the only two genes identifiable in ESTs from multiple species of fish. Following the whole genome duplication, one gene was lost from each locus (resulting in one locus with *Serpinb1 *and another with *Serpinb6*) and each of these loci have since evolved uniquely in descendant fish species (Figure 4). The fish family Polypteridae (bichirs) diverged from teleost fish before the whole genome duplication [22], and as such, a genomic analysis within this family would be predicted to show a single 'primordial' clade B serpin gene locus of *Serpinb1 *and *Serpinb6*.

The identification of a *Serpinb12 *homologue in the *D. rerio *genome suggests that this gene may have evolved significantly earlier than the tetrapod lineage, however, no corroborating ESTs can be found in other fish species. A number of unique clade B serpins have evolved in zebrafish, at both genomic loci, which cannot be found in other fish genomes or EST databases, indicating a more rapid evolution in this fish species. Furthermore, phylogenetic analysis indicates that Danio *Serpinb12 *does not cluster with *Serpinb12 *from other species, but rather with Danio *Serpinb6 *(data not shown), and suggests that the *Danio rerio Serpinb12 *gene may be a case of convergent evolution.

Six genes (*Serpinb3/b4*, *b7*, *b8*, *b9*, *b11 *and *b13*) were gained early along the mammalian branch and the cluster split into two loci, although when these events occurred in relation to each other cannot currently be discerned. The current effort to sequence the platypus (a monotreme) genome, as well as a number of marsupials (the wallaby and opossum), which branched off early in mammalian evolution may shed some light on this.

The analysis of frog *Serpinb12 *clearly shows that it is a 7-exon gene, despite having eight exons in both chickens and mammals. This shows that the 7-exon structure arose first and that the eighth exon was gained in *Serpinb12 *some time after amphibians evolved, but before mammals and birds split. The gain of the CD loop has enabled some members of the clade B serpin family to evolve motifs for secondary functions, without affecting their inhibitory role [23]. The AT-hook motif and nuclear localisation signal (NLS) of chicken MENT [3] are found in the CD-loop, as is the NLS of human bomapin [24]. The extended CD-loop of PAI-2 contains motifs necessary for its suppression of tumour necrosis factor α induced death [23], redox sensitivity [25,26] and cross-linking to cell membranes [27,28] as well as a novel motif for binding to retinoblastoma protein [29].

These analyses suggest that either *Serpinb1 *or *Serpinb6 *is the original clade B serpin gene, and that it was present in the common ancestor of euteleostomi (the bony vertebrates, including both fish and mammals) approximately 450 million years ago. A possible functional orthologue of *Serpinb6 *is evident in the urochordate species *Ciona intestinalis *(sea squirt). However, this gene does not contain any introns, although other serpin genes in the Ciona genome do have introns. This makes it difficult to suggest the Ciona *Serpinb6 *gene as a primordial clade B serpin. Furthermore, the suggestion that clade B serpins evolved from an intronless precursor would also be contrary to a recent study suggesting that they arose from an intron-containing gene [30].

The question of where clade B genes arose may be answered when the genomes of the skate and lamprey are completely sequenced. These species diverged at different times between the sea squirt-human and fish-human common ancestors, and may indicate which gene, *Serpinb1 *or *Serpinb6*, evolved first, and whether or not clade B serpins are descendant from an intronless precursor.

## Conclusion

By investigating the genomes of various vertebrate species, we have shown that the all clade B serpins are descended from a single gene (either *Serpinb1 *or *Serpinb6*) that contained seven exons. The additional exon present in the 8-exon structure appeared in the *Serpinb12 *gene at some point after the divergence of amphibians from amniotes, but before the divergence of birds and mammals. Therefore, both the 7-exon and 8-exon genes evolved at a single locus that split soon after birds and mammals diverged two yield the two loci present in the human genome.

## Methods

The current builds of vertebrate genomes [22,31-34] were accessed through the NCBI and mined using BLAST [35]. The *Xenopus tropicalis *genome was accessed from the Department of Energy Joint Genome Institute. Rabbit genomic contigs were downloaded from the Broad Institute. GENSCAN [36] was also used to predict genes from genomic sequence. Intron/exon boundaries were predicted with Spidey. Alignments were created using ClustalW [37] and manually adjusted with BioEdit (version 6.0.7). Phylogenetic relationships were determined using the Neighbour-Joining method with 1,000 bootstraps and trees were viewed with TreeView [38]. Accession numbers for the sequences identified in this study are given in Table 1.

## Authors' contributions

DK performed the analysis and drafted the manuscript. PIB assisted in design of the study and in drafting of the manuscript.
